# Laser Direct‐Write Sensors on Carbon‐Fiber‐Reinforced Poly‐Ether–Ether–Ketone for Smart Orthopedic Implants

**DOI:** 10.1002/advs.202105499

**Published:** 2022-02-10

**Authors:** Xingjian Hu, Jincai Huang, Yanzhuo Wei, Haiyan Zhao, Shize Lin, Chuxiong Hu, Ze Wang, Zhe Zhao, Xining Zang

**Affiliations:** ^1^ Department of Mechanical Engineering Tsinghua University Beijing 100084 China; ^2^ Key Laboratory for Advanced Materials Processing Technology Ministry of Education Tsinghua University Beijing 100084 China; ^3^ State Key Laboratory of Tribology Tsinghua University Beijing 100084 China; ^4^ Orthopedics Department Beijing Tsinghua Changgung Hospital School of Clinical Medicine Tsinghua University Beijing 100084 China

**Keywords:** CFR‐PEEK, fracture healing assessment, laser annealing, orthopedic implants, strain sensors

## Abstract

Mechanically close‐to‐bone carbon‐fiber‐reinforced poly‐ether–ether–ketone (CFR‐PEEK)‐based orthopedic implants are rising to compete with metal implants, due to their X‐ray transparency, superior biocompatibility, and body‐environment stability. While real‐time strain assessment of implants is crucial for the postsurgery study of fracture union and failure of prostheses, integrating precise and durable sensors on orthopedic implants remains a great challenge. Herein, a laser direct‐write technique is presented to pattern conductive features (minimum sheet resistance <1.7 Ω sq^–1^) on CRF‐PEEK‐based parts, which can act as strain sensors. The as‐fabricated sensors exhibit excellent linearity (*R* ^2^ = 0.997) over the working range (0–2.5% strain). While rigid silicon‐ or metal‐based sensor chips have to be packaged onto flat surfaces, all‐carbon‐based sensors can be written on the complex curved surfaces of CFR‐PEEK joints using a portable laser mounted on a six‐axis robotic manipulator. A wireless transmission prototype is also demonstrated using a Bluetooth module. Such results will allow a wider space to design sensors (and arrays) for detailed loading progressing monitoring and personalized diagnostic applications.

## Graphical abstract

Carbon‐fiber‐reinforced poly‐ether–ether–ketone (CFR‐PEEK) is a rising material to replace metal in orthopedic implants. A laser direct‐write method with multiple degrees of freedom is used to fabricate strain sensors on CFR‐PEEK parts with both lamella and complex structures. Wireless signal transmission is deployed by a Bluetooth module, allowing real‐time monitoring of the detailed loading progressing monitoring and personalized after‐care for patients.

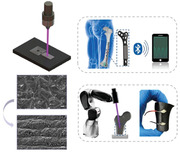

## 1 Introduction

Poly‐ether–ether–ketone (PEEK) arose in the 1990s as a substitute for the metal components and implants of high‐performance thermoplastic polymers, due to its great biocompatibility, heat resistance, X‐ray transparency, chemical inertness, mechanical durability.^[^ ^1^, ^2^ ^]^ With a slight increase in density (<10%), light and strong (3–5 GPa tensile strength) carbon fibers dispersed in the matrix promote the modulus of PEEK (3–4 GPa) to match the value of cortical and cancellous bone (≈18 GPa).^[^ ^3^ ^]^ Therefore, carbo fiber reinforced Poly‐Ether–Ether–Ketone (CFR)‐PEEK has become the most promising material for the next generation of orthopedic implants for joint arthroplasty,^[^ ^4^, ^5^ ^]^ fracture fixation,^[^ ^6^, ^7^ ^]^ spinal cages,^[^ ^8^, ^9^ ^]^ dental implants,^[^ ^10^, ^11^ ^]^ etc.

Directly measuring the in vivo strain of bones is difficult, while probing the strain of the implants instead is more practical to reflect the loading progression and healing status of bones. The current strain sensors integrated on the orthopedic implants are fabricated by time‐consuming and laborious lithography methods and had to be rigidly adhered to implant parts by epoxy.^[^ ^12^, ^13^, ^14^ ^]^ The detrimental nonuniformity of materials and mechanical properties of these assembled implants could potentially decrease their lifespan. In addition, sensors are difficult to affix on the uneven and curved surface of CFR‐PEEK‐based implants, limiting their application in implants with complex structures including joints, tibia plate, and articular interface. Thus, developing directly printed sensors on the CFR‐PEEK matrix with biomechanical uniformity will bring a broad space to design and manufacture sensors with various features and functions targeting different orthopedic applications in the human body.

Laser processing (ablation, annealing, sintering, etc.) has the merits of high precision, noncontact, and less wear over traditional machining methods, which make them desirable in micro‐ and nanoscale fabrications.^[^ ^15^, ^16^ ^]^ Previously, lasers with ultrashort pulses (120 fs at 795 nm, 11 W) ^[^ ^17^ ^]^ or ultrafast scan rates (1–2 m s^‐1^ at 355 nm, 23 W) ^[^ ^18^ ^]^ have been used to ablate CFR‐PEEK with less heat‐affected zones (HAZ), and fiber laser has been applied to texture the CFR‐PEEK surface to improve the friction behavior (1064 nm, 20 W) ^[^ ^19^ ^]^ and lap joining with alloys (1070 nm, 6 kW) ^[^ ^20^ ^]^ of CFR‐PEEK. Rarely any work reports the laser carbonization of CFR‐PEEK.

**Figure 1:**
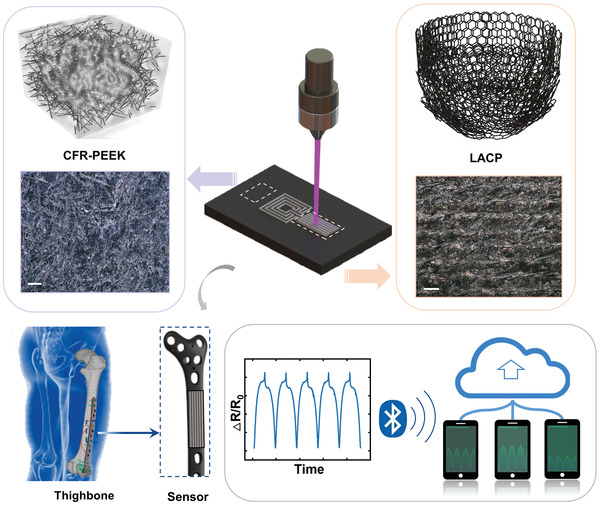
The schematic of direct writing of laser annealed CFR‐PEEK (LACP) based sensor on a thigh bone lamella‐shape implant and its signal transmission with cell phones for continuous outreading of strain via Bluetooth.

In this work, we used a UV nanosecond laser (355 nm, output power 5.5 W at 40 kHz) as a local heat source to directly write devices on the CFR‐PEEK parts (**Figure** **1**). The morphology, chemistry, and conductivity of laser annealed CFR‐PEEK (LACP) are controlled by laser parameters. Using optimized parameters, the sheet resistance of LACP and LAP can be as low as ≈1.7 and ≈3 Ω sq^–1^, respectively. As‐fabricated strain sensors show superior linearity (*R* ^2^ = 0.997) over the loading range (0–2.5% strain). With the aid of a six‐axis robotic arm, we greatly extend the freedom to pattern strain sensor on implants with irregular and complex curved shapes such as hip joints. The sensor on the stress concentrated position of the hip joint prosthesis shows a segmented linear response to strain. Within the range from 0% to 0.29% strain, the gauge factor is 9.198 (*R* ^2^ = 0.997). Furthermore, we integrate the sensor with a Bluetooth module to realize wireless communication with a cellphone. Our work unites the thermal processing of CFR composite materials, manufacturing of sensor with multi‐degree of freedom, and signal conditioning (amplification and conversion) and transmission, which enables a new sensing platform based on CFR‐PEEK. The results are foundations for digitalized medical devices and personalized smart orthopedics.

## 2 Results and Discussion

A 355 nm UV laser was utilized to anneal the CFR‐PEEK under ambient conditions. As shown in Figure S1a,b (Supporting Information), lasing at focus results in the over ablation of CFR‐PEEK and PEEK. Simply lowering the laser power could induce conductive carbonized layers with poor film qualities and weak adhesion to the substrates.^[^ ^21^ ^]^ By contrast, defocusing the laser yields improved layers by spreading the laser beam spot for a more uniform energy distribution that slightly sacrifices the patterning resolution from 40 to 60 µm. Meanwhile, some sample areas can be irradiated multiple times due to the overlapping of enlarged beam spots.

### 2.1 Materials Characterization

The chemistry, morphology, and conductivity of carbonized products were tuned by controlling the laser defocusing distances and scan rates. Referring to the UV–vis spectra of CFR‐PEEK and PEEK, their similar reflectivities at 355 nm indicate the close amounts of UV laser energy absorption (Figure S2, Supporting Information).

**Figure 2:**
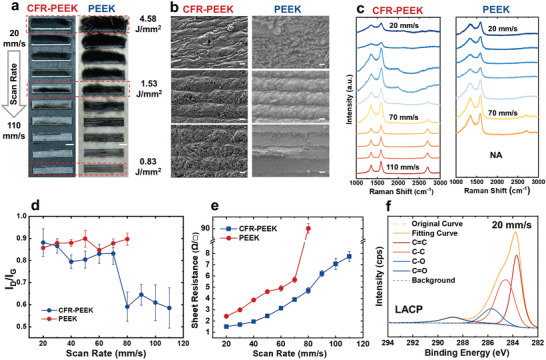
Critical laser parameters. a) Optical photos of LACP and LAP lased by 5.5 W at different laser scan rates ranging from 20 to 110 mm s^‐1^. Scale bar: 2 mm. b) SEM images of LACP and LAP lased using 5.5 W at 20, 60, and 110 mm s^‐1^. c) Raman spectra and d) *I*_D_/*I*_G_ ratios of LACP and LAP at different laser scan rates. e) Sheet resistance comparison of LACP and LAP. f) XPS analysis of LACP produced by 5.5 W laser at 20 mm s^‐1^. Scale bar: 20 µm.

Optical photos (**Figure** **2**a) and scanning electron microscopy (SEM) images (Figure 2b,c) of LACP and LAP under different scan rates are compared to show the morphologies of laser‐induced films. In the case of CFR‐PEEK, the superficial loose carbon black layer gradually reduces and LACP patterns exhibit foamy dense structures with the carbon fibers exposed. A variation of thickness from ≈300 to ≈200 µm was observed when the scan rate changes from 20 mm s^‐1^ (4.58 J mm^‐2^) to 110 mm s‐1 (0.83 J mm^‐2^). While, the microstructures of LAP in PEEK have a dramatic transformation, from flakes to flocculent form. Above 70 mm s^‐1^ (<1.31 J mm^‐2^), LAP samples have more macro defects, and PEEK is almost removed rather than carbonized at a high scan rate due to the unique photochemical effect of the UV laser.^[^ ^18^, ^22^, ^23^ ^]^

Raman spectra are commonly used to characterize carbon materials structures, by mainly measuring the D peak near 1350 cm^–1^, G peak near 1600 cm^–1^, and 2D peak near 2750 cm^–1^. The 2D peak reflects the graphitic stacking, while the D/G peak ratio (*I*_D_/*I*_G_) can be used to evaluate the defects, disordering, localization lengths (i.e., *La*), and *sp* ^2^/*sp* ^3^ content of the carbon product.^[^ ^24^, ^25^, ^26^ ^]^ The Raman spectra of LACP and LAP samples in Figure 2c and their *I*_D_/*I*_G_ ratios in Figure 2d reveal two major schemes of the carbon materials evolution depending on the laser power densities controlled by the scan rates. Above 80 mm s^‐1^ (<1.15 J mm^‐2^) selective bond‐breaking induced ablation dominates the laser‐PEEK interactions, leaving the trenches on the PEEK surface. The strong fluorescence in non‐carbonized PEEK samples overwrite the Raman signature so that LAP samples produced using 90–110 mm s^‐1^ show only broadband backgrounds. In the case of CFR‐PEEK, similar selective ablation is associated with carbon‐fiber annealings and recrystallizations that show significant 2D peaks in Figure 2d. The descending of *I*_2D_/*I*_G_ is accompanied by the increase of *I*_D_/*I*_G_ related to *sp* ^3^ clusters and disorder, as shown on the spectra of LACP samples in Figure 2e. Below the threshold scan rate of 70 mm s^‐1^ (>1.31 J mm^‐2^), 2D peaks are nearly invisible on the Raman spectra of LACP samples. Cross‐compared with the 41.92% *sp* ^2^ C1_s and 40.46% *sp* ^3^ C1_s derived from the XPS spectrum of CFR‐PEEK lased by 20 mm s^−1^ (Figure 2f), the LACP samples produced by 20 mm s^‐1^ should be the highly conductive paracrystalline carbon black structure, illustrated in Figure 1.^[^ ^27^, ^28^ ^]^ Although graphitic stacking is disturbed by the increase of laser energy, the increased carbonization ratio and the decreased C–O and C═O defects in the LACP samples lased at slower scan rates still bring higher conductivities (Figure 2e; Figure S2d, Supporting Information). A similar trend is also observed in LAP samples, where the 2D peak intensity decreases and conductivities increase with an increase in the incident ED (Figure 2c,e). Besides, the repetitive tests of sheet resistance in Figure 2e exhibit great reproducibility of LACP and LAP, especially below 70 mm s^‐1^.

**Figure 3:**
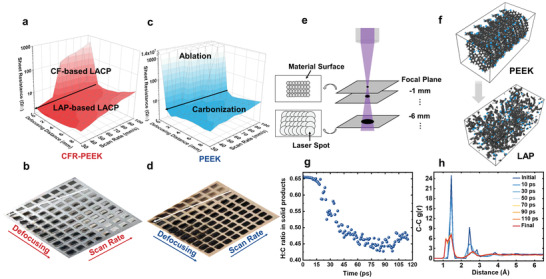
Electronic properties studies of laser annealed products and simulations. Sheet resistance measurements and optical photos of a,b) LACP and c,d) LAP produced at various defocusing distances and scan rates. e) Schematic of multiple lasing in one pass with a large defocusing distance. A, MD simulation of laser annealing PEEK process: f) Configurations of PEEK (before annealing simulation) and LAP (after annealing simulation). g) H:C ratios in the solid products during the annealing process. h) Results of C–C radial pair distribution function g(r) in the annealing process.

The sheet resistance changes of LACP (**Figure** **3**a,b) and LAP (Figure 3c,d) with scan rates and defocusing distances were measured to explore the optimal laser parameters and further study the formation of conductive layers. By tuning parameters, the sheet resistance of LACP suitable for sensor fabrication can vary from ≈1.7 Ω sq^–1^ to ≈18.4 Ω sq^–1^. The minimum resistance appears around defocusing distance = 2 mm. When defocusing distance is below 2 mm, the high ED of the small laser spot leads to overheating and evaporation of PEEK. Conversely, excessive out‐of‐focus value leads to low ED so that PEEK undergoes insufficient carbonization. These two cases have detrimental effects on conductivities. However, the sheet resistance increases monotonically with the scan rate because of less photothermal effects and insufficient pyrolysis of PEEK. It is worth noting that a high scan rate at focus leads to ablation of PEEK while carbonization can still occur at far defocusing distance and high scan rate due to the multiple lasing of enlarged beam spots (see Figure 3e).^[^ ^29^ ^]^ Therefore, there are two regimes in sheet resistance 3D colormap of CFR‐PEEK and PEEK. The ablation area in PEEK is corresponding to the carbon‐fiber‐based (CF‐based) LACP area in CFR‐PEEK. LACP samples in this area are mainly formed by carbon fibers annealings and recrystallizations so that their conductivities are slightly lower than LAP in the same regime. Moreover, the carbonization zone in PEEK is associated with LAP‐based LACP in CFR‐PEEK, wherein samples primarily originate from carbonized PEEK as these two areas share similar sheet resistance.

The laser annealing PEEK process has also been simulated by molecular dynamics (MD). As is shown in Figure 3g, there is a significant decline of the H:C ratio in the early stage due to the dehydrogenation and photothermolysis of PEEK. And the process slows down when more carbon solid frames form and solid products generate. We employed the C–C radial distribution function (RDF), g(r), to quantify the pyrolysis and carbonization process. RDF is a statistical measurement, which describes the chance of finding an atom at a certain distance from a target atom (see Figure 3h). In the beginning, the RDF of PEEK has four distinct peaks, which correspond to the distance of 1.45, 2.45, 2.85, and 3.85 Å from the highlighted carbon atom (Figure S3a. Supporting Information). The 1st peak represents C–C single bonds, and the 2nd and 3rd peaks are indications of the benzene ring. As the heating process continues, the intensity of the 1st peak declines, and the 2nd and 3rd peaks are combined into a new peak located at 2.65 Å. These indicate the breakdown of the benzene rings and the formation of amorphous carbon, which is quite different from the RDF of single‐layer graphene.^[^ ^30^ ^]^ Another new peak appears before the original 1st peak, which represents the gas molecules in the final products such as C_2_H_2_. The gradually fading characteristic peaks of products in XRD spectra (Figure S3b, Supporting Information) also prove the pyrolysis of PEEK during the reaction.

### 2.2 Sensors Characterization

**Figure 4:**
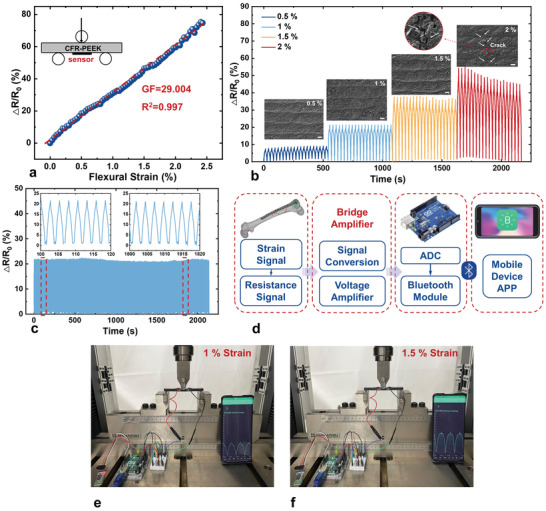
An LACP‐based strain sensor and a wireless transmission prototype. a) Relative resistance change of an LACP‐based sensor responding to the continuous variation of flexural strain. b) Resistance responses and SEM images of the sensor under various external flexural strains (0.5%, 1%, 1.5%, and 2%, ≈27 s cycle^‐1^, scale bar: 20 µm). c) Cyclic test of the sensor at 1% for ≥800 cycles (≈2.4 s cycle^‐1^). d) Block diagram of the Bluetooth wireless transmission system. e,f) Real‐time data display via Bluetooth wireless transmission test at 1% and 1.5% strain.

To mimic the in‐vivo loading progression of CFR‐PEEK orthopedic implants, three‐point bending tests have been performed, and the flexural strain of implants is reflected by resistance variations of the LACP‐based sensor. The sensitivity experiment has been conducted to calibrate the working range and linearity of LACP‐based sensors (**Figure** **4**a). The gauge factor (*GF*) is determined by:

(1) $$GF=\frac{\Delta R}{R_{0}\varepsilon_{F}}$$

in which △*R* and *R*_0_ represent the absolute value of resistance increment and the initial resistance, and *ε*_F_ is the flexural strain. According to the experiment, the CFR‐PEEK sample can bear a maximum flexural strain of ≈2.5%. The △*R*/*R*_0_ of the LACP‐based sensor is in positive proposition to the applied strain and shows great linearity over the whole working range with the *GF* = 29.004 and *R* ^2^ = 0.997. As is shown in Figure 4b, it is noted that the output signals of the sensor are stable when *ε*_F_ ≤ 1.5%, but the maximum △*R*/*R*_0_ shows obvious damping at *ε*_F_ = 2% after several cycles. According to the SEM images in the inserts of Figure 4b, there are no distinct morphological changes when *ε*_F_ increases from 0.5% to 1.5%. However, microcracks and a slight swell of conductive layers were observed after 2%, and these morphological conversions cannot recover due to the plastic deformation of CFR‐PEEK samples. Therefore, it can be concluded that the resistance variance is mainly attributed to microcrack generation and thickness alteration of conductive LACP layers. The performance stability of the sensor is also proved by >800 cycles of dynamic bending‐releasing between 0 and 1% strain (Figure 4c). The waveforms of all cycles are almost identical, and the maximum value is consistent with that in Figure 4a,b, indicating the good reliability and reproducibility of the sensor. The LACP‐based sensors can also transmit the signal wirelessly to mobile devices by connecting with the Bluetooth module and peripheral circuits (Figure 4d). As is shown in Figure 4e,f, a mobile device such as a cell phone can display the real‐time signal collected by the sensor from the CFR‐PEEK implant. And it is worth noting that the output waveforms are identical to those in wired signal transmission. In the future, the whole wireless transmission system could be miniaturized and fabricated into a flexible printed circuit (FPC), which could be easily mounted and packaged on the implant.

The LACP‐based sensors on CFR‐PEEK can also act as compressive sensors or tensile sensors. As shown in Figure S4 (Supporting Information), the trends of resistance over the strain in compressive sensors and tensile sensors are opposite. However, they all have three quasi‐linear regions in the low, medium, and high strain range, which are different from the results of bending tests. As for the compressive sensor, the gauge factor decreases as the strain increases. And it is noteworthy that the sensor is dramatically sensitive to tiny compressive strains, with a gauge factor of >160 in the first linear region. In comparison, the gauge factor of the tensile sensor is positively related to the strain, ranging from 2.674 to 32.975. These results reveal that the LACP‐based sensors also have great potential in implants that are primarily subjected to compressive and tensile stresses such as articular interfaces, spinal cages, and dental implants.

**Figure 5:**
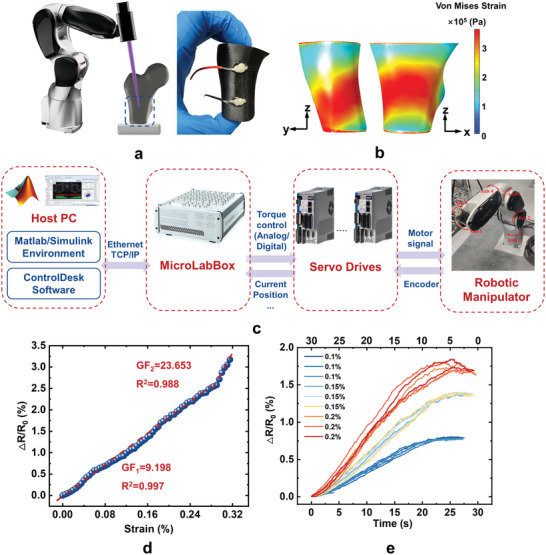
Printing a strain sensor on a complex surface. a) Schematic of LACP‐based sensor fabrication on CFR‐PEEK‐based thighbone prosthesis model with the aid of a six‐axis robotic manipulator. b) Von Mises strain contour of the model under compression simulation by FEA. c) System‐level block diagram of a robotic manipulator control system. d) The relative resistance change of the LACP‐based sensor with the continuous variation of compressive strain. e) Hysteretic curves of the sensor cyclic test at 0.1%, 0.15%, and 0.2% (≈60 s cycle^‐1^).

Considering that the surfaces of real implants or prostheses are usually complex, we fabricated an LACP‐based sensor on a CFR‐PEEK thighbone prosthesis model with the aid of a six‐axis robotic manipulator (**Figure** **5**a). The sensor pattern can fit well to the curve surface of the prosthesis model via path planning of the robotic manipulator. Before the compressive experiment, finite element analysis (FEA) simulation was performed to reveal the areas of stress concentration on the model during the loading process (Figure 5b) and determines the printing location (Figure 5c). The sensitivity test reveals that the LACP‐based sensor on this prosthesis has segmented linear regimes divided by compressive strain as ≈0.29%. We also validated the reusability of the sensor in the first linear response regime (see Figure 5d), and the sensor can maintain stable performance during repeated loading–releasing processes (Figure 5e). The LACP‐based sensor fabricated by the robotic arm is slightly different from that fabricated by the commercial laser apparatus in resistance (≈10 times higher) and microstructure (more porous). It is because of the difficulty in exact fixing of prosthesis on the origin of coordinates and the simpler laser module mounted on the robotic arm compared with the commercial apparatus. Moreover, the surface features of the prosthesis model may be slightly different from the original 3D printing digital model. Therefore, it is crucial to increase the sensor quality and processing stability by robotic arms in the future.

## 3 Conclusion

In summary, we demonstrate a direct writing method to fabricate an in situ strain sensor on CFR‐PEEK‐based orthopedic implant parts for the first time. The laser annealing mechanism of CFR‐PEEK and PEEK has been studied, and optimized laser processing parameters were found to achieve low sheet resistance down to ≈1.7 Ω sq^–1^. An in situ flexural strain sensor was fabricated with excellent linearity (*R* ^2 ^= 0.997) over the working range and great stability. The sensor can also transmit signals wirelessly to mobile devices by connecting with a Bluetooth module. Furthermore, with the help of a six‐axis robotic manipulator, we can fabricate sensors on various CFR‐PEEK‐based implants or prostheses with complex free‐form surfaces. The production of LACP and LACP‐based strain sensors paves the way for detailed loading progressing monitoring and personalized diagnostic applications. PEEK has been widely used in clinical applications, and CFR‐PEEK has been approved for medical use by FDA and CE. In future, we will conduct biocompatibility^[^ ^31^ ^]^ and cell adhesion tests of LACP and LAP. The rough surfaces of laser engineered features could potentially induce biofouling that is detrimental. Thus, packaging methods and loading tests of packaged devices are essential for in vivo clinical study.

## 4 Experimental Section

### LACP and LAP Production

A 355 nm DPSS Nd:YVO_4_ pulsed laser (Bellin Laser) was used with an average output power = 5.5 W and repetition frequency = 40 kHz. The pulse duration is ≈25 ns with ≈16 µm spot size at focus. Samples were fabricated on 6 mm‐thick PEEK and CFR‐PEEK (PEEK + 30% short PAN‐based carbon fibers, JUNHUA PEEK) plates at 2 mm above the laser focal plane in one pass. The inherent electrical conductivity of the PAN‐based carbon fiber was ≈1 S cm^–1^.^[^ ^32^, ^33^ ^]^ The plates were cleaned with ethanol before processing, and laser irradiation was conducted in the ambient environment. The energy density (ED) of laser can be defined as:^[^ ^34^ ^]^

(2) $$ED=\frac{P_{\mathrm{avg}}}{V_{\mathrm{scan}}\emptyset_{\mathrm{spot}}}$$

where *P*_avg_ is the laser average power (W), *V*_scan_ is the scan rate (mm s^‐1^), and ∅_spot_ is the spot diameter of the laser beam (mm).

### Material Characterization

Micro‐optical photos were taken by Keyence VHX‐6000. SEM was performed on Zeiss Gemini 300 with a 5 keV electron beam. Raman spectroscopy was conducted on Horiba Lab RAM HR Evolution with a 532 nm laser source. X‐ray photoelectron spectroscopy was conducted on Thermo Fisher ESCALAB 250Xi with C1_s as target regions. The sheet resistance of samples was measured by Keysight B2912A precision source meter unit (SMU) on Cascade EPS150FA probe station, using the four‐probe method (also known as the Kelvin technique, see Figure S5, Supporting Information). The sheet resistance can be calculated in the equation below:^[^ ^35^ ^]^

(3) $$R_{S}=C\frac{\pi }{\ln\left(2\right)}\frac{\Delta V}{I}\approx 4.53236\cdot C\frac{\Delta V}{I}$$

where *C* is the geometric correction factor. For a 4 mm × 2 mm square sample, *C* = 0.4292.

### Sensor Production and Characterization

The CFR‐PEEK plates were machined into specific shapes (see Figure S6, Supporting Information) according to ISO 527‐2:2012 and ISO 178:2019. The dimension of a printed sensor was 25 mm × 5 mm. In most cases, the sensors were lased at 60 mm s^‐1^ scan rate and 2 mm defocusing distance considering patterning precision (clear pattern outline), sensor performance (excellent linearity and sensitivity), and connection with telemetry circuitry (low complexity in signal conversion). The wires were connected to the sensor by the conductive silver paste. After the paste dried, acrylic resins were coated on the joints for fixation and protection. The three‐point bending, compressive, and tensile tests were all performed on the same Shimadzu AGX‐V universal material tester with different customized clamps. The electrical signals were measured by Keysight B2912A SMU, which also served as a 1 V voltage source. Considering that the new CFR‐PEEK samples had residual stress because of extrusion molding and machining, the resistance variance of sensors on these samples was usually irregular in the early loading stage. To make different samples comparable, 0.3% flexural strain was applied and released to precondition all the new samples.

### Signal Wireless Transmission

There were four main modules in the wireless transmission system. The LACP‐based sensor was connected with Arduino UNO microcontroller via a Wheatstone bridge module. The Bluetooth module was also mounted on the Arduino board. The sensor first converted the flexural strain of CFR‐PEEK implants into resistance variation, which underwent signal amplification (gain factor: ≈1.21) and was reflected by the output voltage of the Wheatstone bridge. Then, the signal further underwent analog to digital conversion (ADC) in Arduino UNO, and it was transmitted wirelessly via Bluetooth module. Finally, Bluetooth‐enabled mobile devices could receive and display real‐time data from the LACP‐based sensor.

### Sensor Fabrication on Free‐Form Surfaces

A 15 W 455 nm compact semiconductor laser module was employed and mounted on a 6‐DOF robotic manipulator (COMAU Racer3). The vertex coordinates and normals were extracted from the prosthesis model for the focal length control. As for the robotic manipulator system, the host PC built the control model designed for path tracking and communicated with dSPACE ds1202 MicroLabBox (5 kHz) with TCP/IP. Then, the MicroLabBox translated the built model into real‐time torque control signal received by Kollmorgen AKD‐P servo drives. Finally, the servo drives controlled the motors of the robotic manipulator. Meanwhile, the manipulator could also feedback information such as current position to the MicroLabBox via servo drives. The finite element analysis (FEA) of the half thighbone model was conducted on COMSOL Multiphysics. The FEA simulation was designed based on the real compressive experiment on the universal material tester. The model is shown in Figure S7 (Supporting Information). In the model, the bottom plate was fixed, and the top plate was loaded with 200 N along the negative direction of the *z*‐axis. The CFR‐PEEK‐based half thighbone prosthesis model was made by 3D printing (apparatus: WH3D HT220, base material: CFR‐PEEK). After sensor fabrication, the compressive experiment was performed on Shimadzu AGX‐V universal material tester.

## Conflict of Interest

The authors declare no conflict of interest.

## Supporting information

Supporting Information

## Data Availability

The data that support the findings of this study are available from the corresponding author upon reasonable request.
