# Supplementary material for: Laser Direct‐Write Sensors on Carbon‐Fiber‐Reinforced Poly‐Ether–Ether–Ketone for Smart Orthopedic Implants
Source: Adv Sci (Weinh). 2022 Feb 10;9(11):2105499. doi: 10.1002/advs.202105499 (PMC9009116; doi:10.1002/advs.202105499)
Supplement: Supplementary file 1 — Supporting Information [file ADVS-9-2105499-s001.pdf]

ADVANCED SCIENCE
Open Access

Supporting Information

for *Adv. Sci.*, DOI 10.1002/advs.202105499

Laser Direct-Write Sensors on Carbon-Fiber-Reinforced Poly-Ether–Ether–Ketone for Smart Orthopedic Implants

Xingjian Hu, Jincai Huang, Yanzhuo Wei, Haiyan Zhao, Shize Lin, Chuxiong Hu, Ze Wang, Zhe Zhao* and Xining Zang**

ADVANCED SCIENCE
Open Access

Supporting Information

for *Adv. Sci.*, DOI: 10.1002/advs.202105499

Laser Direct-Write Sensors on Carbon-Fiber-Reinforced Polyetheretherketone (CFR-PEEK) for Smart Orthopedic Implants

Xingjian Hu, Jincai Huang, Yanzhuo Wei, Haiyang Zhao, Shize Lin, Chuxiong Hu, Ze Wang, Zhe Zhao*, Xining Zang**

Supporting Information

Laser Direct-Write Sensors on Carbon-Fiber-Reinforced Polyetheretherketone (CFR-PEEK) for Smart Orthopedic Implants

Xingjian Hu, Jincai Huang, Yanzhuo Wei, Haiyang Zhao, Shize Lin, Chuxiong Hu, Ze Wang, Zhe Zhao*, Xining Zang**

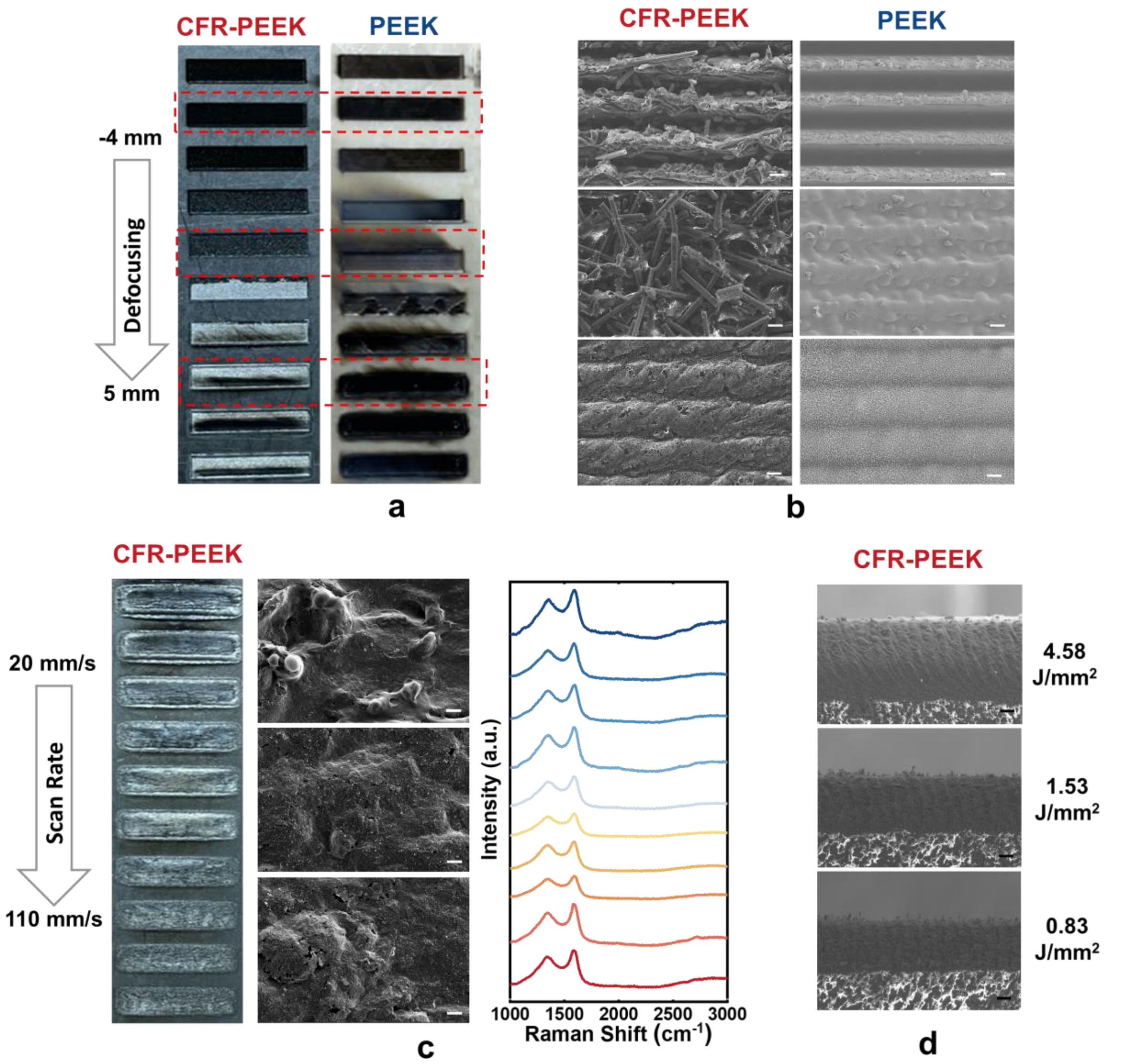

Figure S1. a) Optical photos and b) SEM images of LACP and LAP under different UV laser defocusing distances. c) Optical photos, SEM images, and Raman spectra of LACP irradiated by CO_2 laser with different scan rates. d) SEM images of cross-section views of LACP lased at 20 mm/s, 60 mm/s, and 110 mm/s (defocusing distance: 2 mm). Scale bar: 20 µm.

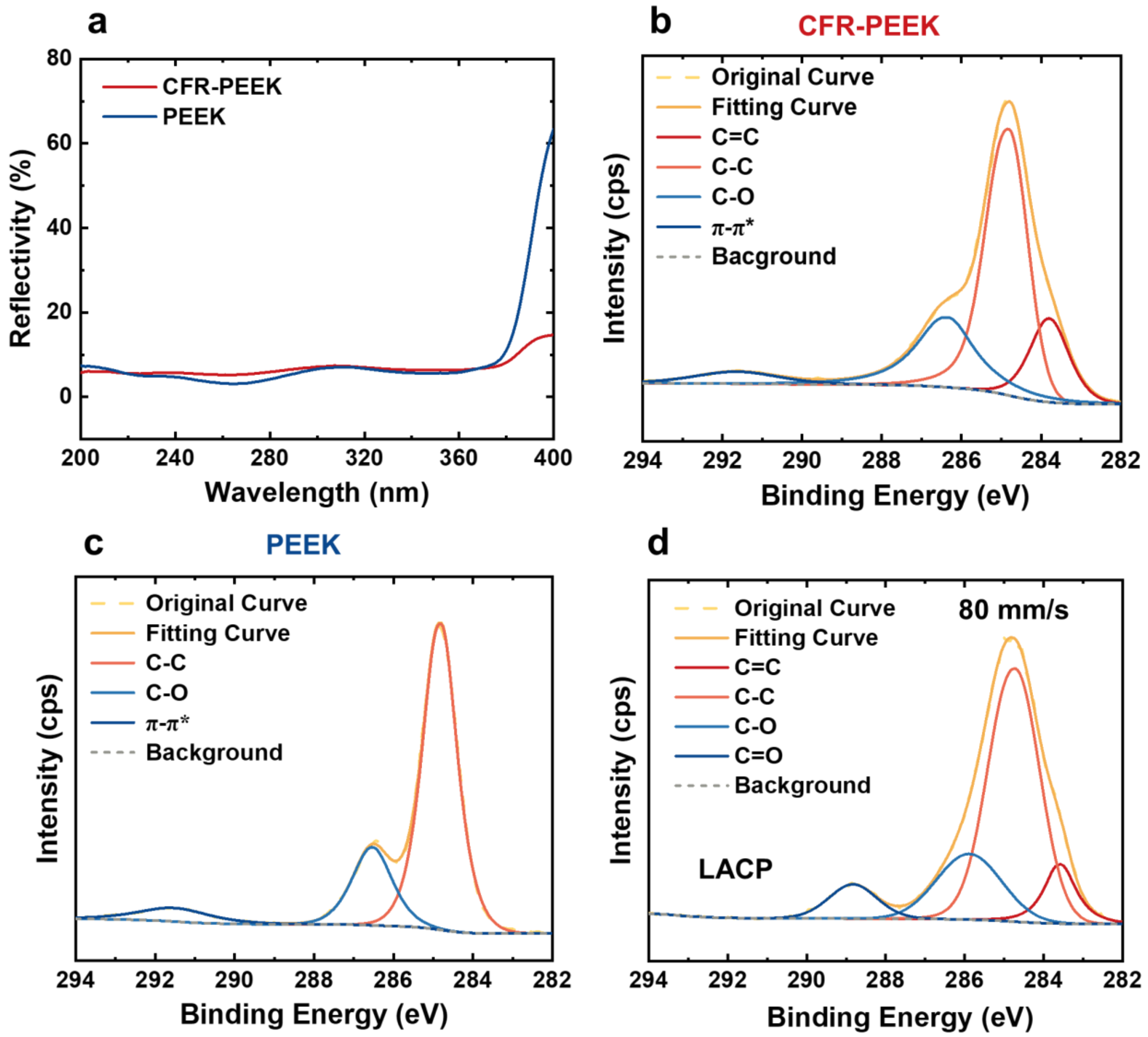

Figure S2. a) Reflectivity of original CFR-PEEK and PEEK in UV-Vis spectrophotometry. XPS analysis of original b) CFR-PEEK, c) PEEK, and d) LACP produced lased at scan rate = 80 mm/s and defocusing distance = 2 mm.

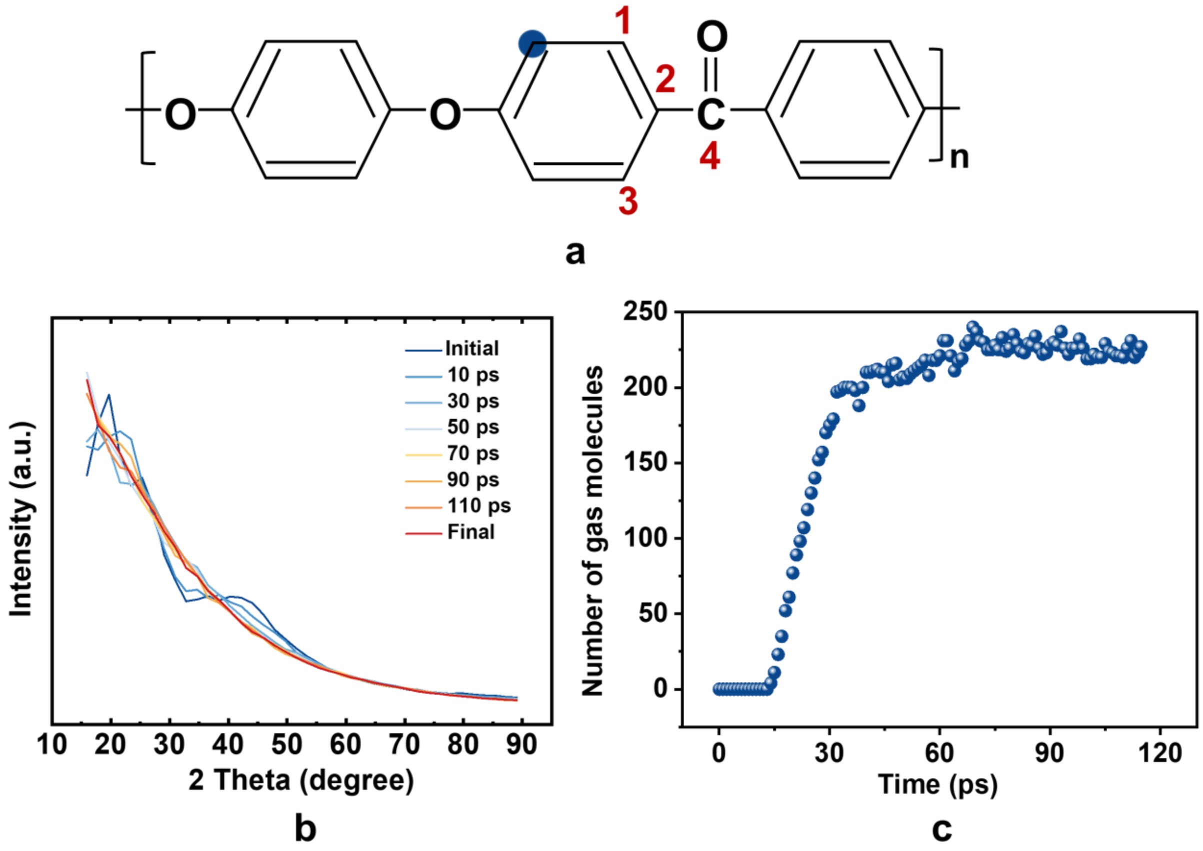

Figure S3. Molecular dynamics simulation results of laser annealing PEEK. a) Structural formula of PEEK and the characteristic position of atoms in RDF. b) XRD spectra of products and c) the number of gas molecules during the laser annealing process.

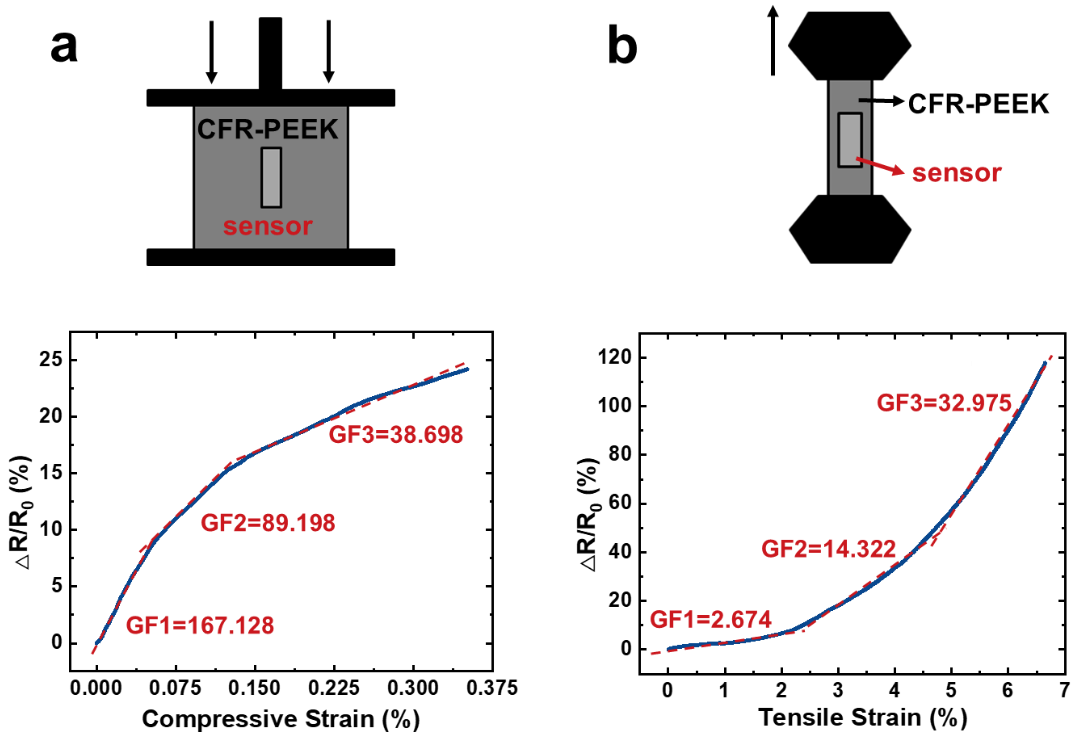

Figure S4. Schematics and characterization of LACP-based a) compressive sensor, and b) tensile sensor.

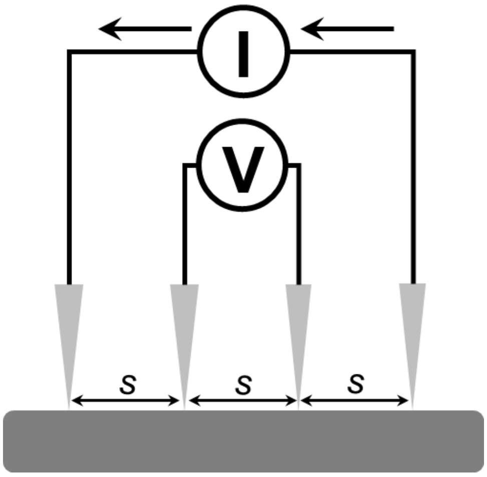

Figure S5. Schematic of four-probe method (Kelvin technique) for sheet resistance measurement, and probe spacing (*S*) is 1 mm.

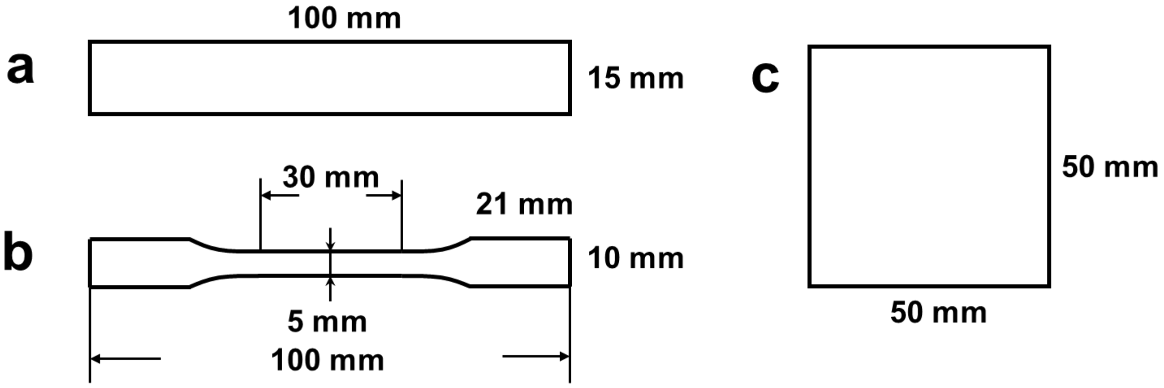

Figure S6. Sample configurations of a) flexural strain, b) tensile strain, and c) compressive strain sensors. Thickness: 6 mm.

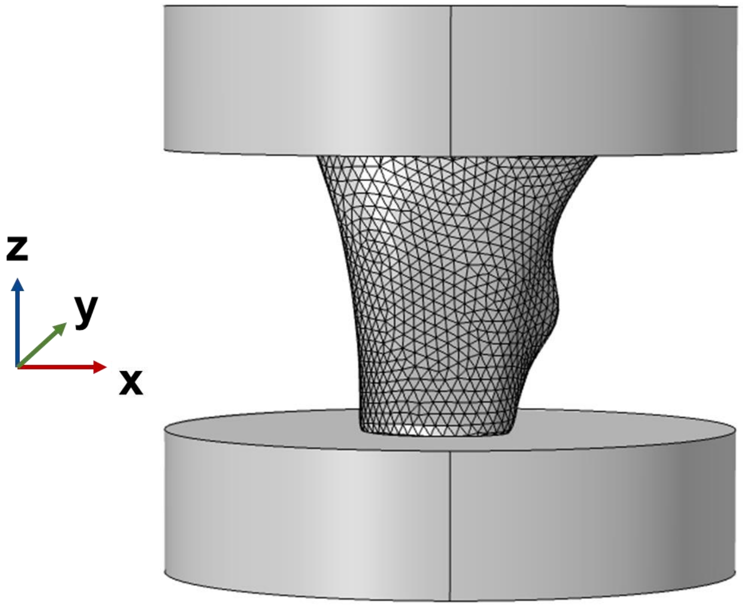

Figure S7. FEA model. The bottom plate is fixed and the top plate is loaded with 200 N (compression).

Table S1. Information and Properties of PEEK [a)]

Molecular structure	Origin structure	Density [g cm^{-3}]	Molecular weight of repeat unit [g mol^{-1}]	Molecular weight	Thermal conductivity [W m^{-1} K^{-1}]	Coefficient of thermal expansion [10^{-5} ℃$^{-1}$]
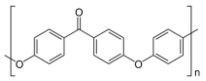	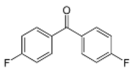	1.3 ± 0.01	288.3	288.3n (n ≈ 4000)	0.28	4.7

[a)] The above information is obtained from the datasheet of PEEK provided by the material supplier (JUNHUA PEEK, China), and the data of thermal properties is proved by the literature.[1,2]

Table S2. Material properties in the FEA model

Items	Material type	Density [kg m^{-3}]	Young's modulus [Pa]	Poisson's ratio
upper/bottom compressive plates	stainless steel[a)]	8000	1.9×10^{11}	0.27
half thighbone model	CFR-PEEK[b)]	1400	2.3×10^{10}	0.35

[a)]The material properties of stainless steel are obtained from the literature;[3,4] [b)]The material properties of CFR-PEEK are obtained from the datasheet provided by the material supplier (JUNHUA PEEK, China), and the data of mechanical properties is proved by the literature.[5,6]

References:

[1] G. Skirbutis, A. Dzingutė, V. Masiliūnaitė, G. Šulcaitė, J. Žilinskas, *Stomatologija* **2017**, *19*, 19.

[2] S. X. Lu, P. Cebe, M. Capel, *Polymer* **1996**, *37*, 2999.

[3] W. Köster, H. Franz, *Metallurgical reviews* **1961**, *6*, 1.

[4] Z. Chen, U. Gandhi, J. Lee, R. H. Wagoner, *J. Mater. Process. Technol.* **2016**, *227*, 227.

[5] C. S. Li, C. Vannabouathong, S. Sprague, M. Bhandari, *Clin. Med. Insights Arthritis Musculoskelet. Disord.* **2015**, *8*, 33.

[6] J. R. Sarot, C. M. Contar, A. C. Cruz, R. de Souza Magini, *J. Mater. Sci. Mater. Med.* **2010**, *21*, 2079.
